# A motivation model for interaction between parent and child based on the need for relatedness

**DOI:** 10.3389/fpsyg.2013.00618

**Published:** 2013-09-12

**Authors:** Masaki Ogino, Akihiko Nishikawa, Minoru Asada

**Affiliations:** ^1^Department of Informatics, Kansai UniversityOsaka, Japan; ^2^Department of Adaptive Machine Systems, Graduate School of Engineering, Osaka UniversityOsaka, Japan

**Keywords:** intrinsic motivation, relatedness, interaction, emotion

## Abstract

In parent-child communication, emotions are evoked by various types of intrinsic and extrinsic motivation. Those emotions encourage actions that promote more interactions. We present a motivation model of infant-caregiver interactions, in which relatedness, one of the most important basic psychological needs, is a variable that increases with experiences of emotion sharing. Besides being an important factor of pleasure, relatedness is a meta-factor that affects other factors such as stress and emotional mirroring. The proposed model is implemented in an artificial agent equipped with a system to recognize gestures and facial expressions. The baby-like agent successfully interacts with an actual human and adversely reacts when the caregiver suddenly ceases facial expressions, similar to the “still-face paradigm” demonstrated by infants in psychological experiments. In the simulation experiment, two agents, each controlled by the proposed motivation model, show relatedness-dependent emotional communication that mimics actual human communication.

## 1. Introduction

Humans acquire knowledge and skills voluntarily by interacting with the environment. This voluntary learning process is driven by intrinsic motivation, which embodies curiosity and interest. By contrast, extrinsic motivation results in rewards such as food. White (1959) proposed that the intrinsic desire to interact with the environment and others underlies human exploratory behavior. Intrinsic motivation encourages individuals to seek novelty, uncertainty, and complexity (Berlyne, 1960). According to the self-determination theory of Ryan and Deci (2000), humans have three inherent fundamental needs: autonomy, competence, and relatedness. Autonomy is the perception that one's behavior is compatible with one's approval. Competence is fulfilled when expected or desired results are achieved. Relatedness is gained when one senses a close relationship with others. Ryan and Deci insist that these fundamental needs and individual differences are shaped by the social context.

Fundamental needs are closely related to emotions. Reis et al. (2000) showed that satisfaction levels of fundamental needs are correlated with emotional evaluation indices. Interestingly, while the satisfaction levels of autonomy and competence correlate with both positive and negative emotions, the relatedness level correlates only with positive emotions. Closely related persons evoke more emotions than strangers. If relatedness is not satisfied, unpleasant emotions are not necessarily evoked, but people sense discomfort when an expected reaction is not delivered by the related person. Thus, compared with the other two needs, relatedness exerts a more complicated effect on emotions.

The need for relatedness becomes apparent from the early stage of infant development. Still-face paradigm experiments have shown that infants are socially sensitive to others (Adamson and Frick, 2003; Striano, 2004). In these experiments, the caregiver suddenly ceases normal interaction with the infant and shows a still face. Throughout this phase, the caregiver reduces the number and extent of positive activities, such as smiles or attention. Infants react to this behavior with restore reactions such as clapping or reaching to the caregiver to draw their attention. The reactions shown by infants depend on their development stages (Adamson and Frick, 2003). These experiments show that infants are motivated to establish relatedness with others and that they require attachment to others.

Several studies in cognitive developmental robotics (Asada et al., 2009) have sought to understand initial communication by computational models (Ogino et al., 2007; Watanabe et al., 2007). However, these studies focused on acquiring communicative actions, rather than the factors that motivate communication. The mechanism that encourages an agent to behave according to internal discipline rather than external reward has been identified as intrinsic motivation (Barto et al., 2004; Oudeyer et al., 2007). However, intrinsic motivation studies continue to adopt self-learning tasks such as skill acquisition. The question remains: how do intrinsic motivation mechanisms promote communicative interactions?

This paper proposes a motivation model of early communication between an infant and his/her parent, in which the need for relatedness triggers emotional change and behavior learning. The proposed model aims for dynamic interaction between two agents who estimate each other's the internal state. Throughout the interaction, an interpersonal relationship is established in which approaching and sharing another's emotion encourages interest and relatedness to him/her, alters emotional states, and promotes mutual behavior. While relatedness directly and indirectly affects the emotional state of an agent, it also changes the reward for action selection. We consider that the network of dopamine neurons plays an important role in activating communication. Dopamine neurons are known to code the prediction error for reward in reinforcement learning (Schultz, 1998). In robotics, (Kaplan and Oudeyer, 2007) hypothesized that dopamine neurons encode signals for encouraging behavior that decreases the prediction error. Recent studies reveal that dopamine neurons are activated not only by explicit reward but also by novel signals that are not directly related to these rewards (Dayan and Balleine, 2002; Kakade and Dayan, 2002). This indicates that dopamine neurons play an important role in intrinsic motivation. Dopamine neurons are also associated with emotional reactions in the amygdala (Phillips et al., 2010). From these neuroscience findings, it is reasonable to consider a model in which a variable corresponding to dopamine neurons mediates emotional change and behavior. In parent infant interactions, the activation of dopamine neurons will arouse the infant's interest, and the parent will act to maintain this interest.

## 2. Materials and methods

### 2.1. Motivation model of parent–infant interaction

**Figure 1:**
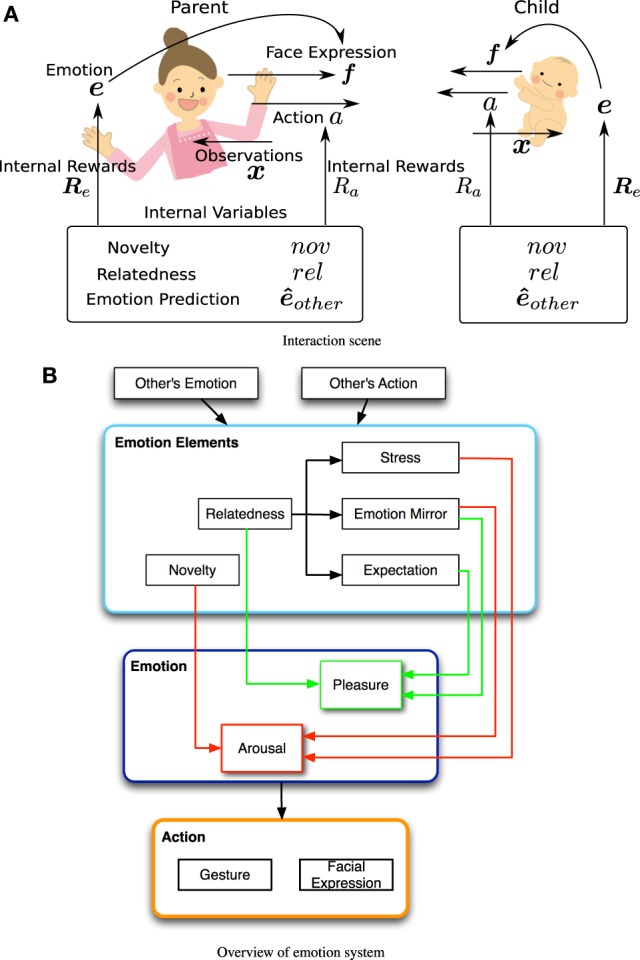
**(A)** Variables used in the proposed model in an interaction situation. **(B)** Overview of the emotion system.

In the communication situation of this study, an infant attentively interacts with his/her caregiver and displays emotional facial expressions such as laughing and crying. The interaction situation and variables used in the proposed model are shown in Figure 1A. The infant and the parent update their internal state, *e*, based on the observed information, *x*, and output their facial expressions, *f*, and actions, *a*. The facial expressions and actions are assumed to be produced and observed independently. The facial expressions are based on the agent's internal state, *e*, which partly depends on the facial expressions of the other agent, *e*_other_. We suppose that both agents (parent and child) possess the same emotional system, comprising *emotional elements, emotion*, and *action selection* modules (Figure 1B). The *emotional elements* module contains two main elements for intrinsic needs, *Novelty* and *Relatedness*, and other three sub-elements, *Stress, Emotion Mirror* and *Expectation*. The value of each element is determined by the other's facial expressions and actions. The emotion elements are used to compute the current emotional state of the agent in the *emotion module*. Finally, in the *action module*, the reward value is evaluated from the emotional elements (pleasure and arousal), and gesture and facial expressions are selected. The following subsections describe the mechanisms of the internal state.

#### 2.1.1. Emotion

Russell (1980) proposed that all emotional states lie within a two-dimensional space comprising an arousal–sleep axis and a pleasure–unpleasure axis. Following Russell's model, we define the emotional state *e* as a vector of arousal and pleasure elements.

(1) $$e(t)=\left(\begin{array}{c} e^{\text{Arousal}}(t)\\ e^{\text{Pleasure}}(t) \end{array}\right)=\left(\begin{array}{c} e^{A}(t)\\ e^{P}(t) \end{array}\right)$$

The emotional state is updated by the reward, ***R***_*e*_, as follows;

(2) $$e(t+1)=e(t)+\eta (R_{e}(t)-e(t)).$$

The elements of the reward corresponding to arousal and pleasure, *R*^*A*^_*e*_ · *R*^*P*^_*e*_, are composed of various psychological factors—novelty, relatedness, emotional contagion, and expectancy—denoted nov, rel, *e*_const_, str, and *E*_grad_, respectively.

(3) $$R_{e}^{A}(t)=\alpha^{A}\text{nov}(t)+\beta^{A}\text{str}(t)+\gamma e_{\text{cont}}^{A}(t)$$

(4) $$R_{e}^{P}(t)=\alpha^{P}\text{rel}(t)+\beta^{P}E_{\text{grad}}(t)+\gamma e_{\text{cont}}^{P}(t).$$

The novelty, nov, indicates the degree of interest in novel surrounding objects, and it is defined as

(5) nov(t)=1/(1+exp(−m(I(t)−θ)),

where *I*(*t*) is information gain, and *m* and θ are constants. The information gain, *I*(*t*), is based on a state transition model constructed by the agent's observations,

(6) I(t)=−logp(s(t+1)∣s(t)).

Stern (1985) proposed that the emotional attunement of a parent is important in establishing a parent–child relationship. Such emotional attunement is thought to be necessary for the sharing mind states. Thus, we assume that the relatedness variable, *rel*, depends on the synchronization of emotional states:

(7) rel(t)=(1−μ)rel(t−1)+νsim(t),

where μ and ν are constants. sim is the emotional similarity, i.e., the extent to which other's emotional states are shared between the agents. sim is the inner product of the self and other's emotion vectors:

(8) $$\text{sim}(t)=e(t)e_{\text{other}}(t).$$

As suggested by fMRI experiments (Singer et al., 2004), humans possess an emotional mirror system. A person's emotional state is slightly altered by the perceived emotional state of another. In this paper, the variable for emotional contagion variable, *e*_cont_, is the product of relatedness and the emotional state of the other:

(9) $$e_{\text{cont}}(t)=\text{rel}(t)e_{\text{other}}(t).$$

Note that the emotional contagion increases with increasing degree of relatedness.

When a parent is unwilling to relate to his/her infant, the heart rate of the infant increases, and the infant's gaze is averted from the parent, apparently because the infant is temporarily aroused by the stress of communication failure (Field, 1981). In our model, the stress variable increases when emotional sharing with the related person fails; that is

(10) str(t)=rel(t)exp(−σsim(t)).

where σ is a positive constant.

While emotional contagion and stress cause temporary effects, the impact of emotional expectancy is long lasting. For example, pleasure is enhanced when one's action appears to please another. Thus, we define emotional expectancy as temporal gradient of expected pleasure, defined by multiplying the action selection probability by the pleasure of the other at the present and preceding moments, and taking their difference.

(11) $$E_{\text{grad}}(t)=\text{rel}(t)\left(p_{a}(t)e_{\text{other}}^{P}(t)-p_{a}(t-t_{b})e_{\text{other}}^{P}(t-t_{b})\right)$$

The emotional expectancy is large when the action selection probability and the pleasure emotion increase together. Emotional expectancy is also affected by relatedness.

#### 2.1.2. Motivation mechanism for action

Dopamine neurons in the midbrain are considered to encode values; they are activated and suppressed in desirable and undesirable situations, respectively. However, some dopamine neurons have recently been reported as activated even in undesirable situations. Bromberg-Martin et al. (2010) proposed that dopamine neurons encode either motivational value or motivational salience. Thus, we model two classes of dopamine neurons, as follows.

Dopamine neurons belonging to the first class, encoding a motivational value, are projected from the basal ganglia and contribute to the exploratory and evaluative learning of whether the current situation is desirable/undesirable. In infant–parent communication, actions that attract the infant's interest and establish relatedness will score high motivational value. Thus, we suppose that the first class of dopamine neurons encodes the other's arousal emotion and relatedness,

(12) $$R_{a}^{\text{Value}}(t)={\hat{e}}_{\text{other}}^{A}(t)+\omega \text{rel}(t),$$

where ω is a positive constant.

Dopamine neurons belonging to the second class, motivational salience, are projected from the amygdala. The neurons contribute to the learning of motivationally important events that may not be related to reward and are thought to aid attention and working memory. We suppose that the second class of dopamine neurons encodes the arousal emotion

(13) $$R_{a}^{\text{Salience}}(t)=e^{A}(t).$$

Both rewards are summed to give the total reward

(14) $$R_{a}(t)=\rho R_{a}^{\text{Value}}(t)+(1-\rho )R_{a}^{\text{Salience}}(t),$$

where ρ is a weighting constant (0 ≤ ρ ≤1). As ρ increases, an agent acts upon predictions of the other's emotional state. If ρ is small, an agent acts more upon its own emotional response.

Reinforcement learning is used to update the action policy. Although various sensor information is important in actual communication, here we consider actions alone. When an action *a* yields a reward *R*_*a*_, the corresponding action value function ${\hat{R}}_{a}$ is updated as

(15) $${\hat{R}}_{a}(t+1)\leftarrow {\hat{R}}_{a}(t)+\eta_{R_{a}}\left(R_{a}(t)-{\hat{R}}_{a}(t)\right),$$

where η_*R*_*a*__ is a learning coefficient.

The parent agent adopts an ε-greedy policy. That is, with probability ε, the parent agent selects the highest-valued action, ${\hat{R}}_{a}$, among its own action repertoire, and otherwise chooses random actions,

(16) $$\pi_{\text{parent}}(t)=\{\begin{array}{ll} \text{random action} & \left(\zeta <\varepsilon \right)\\ \arg\;\max_{a}{\hat{R}}_{a}(t) & \left(\text{otherwise}\right) \end{array},$$

where ζ is randomly drawn from a uniform distribution in the interval [0, 1].

The actions performed by the infant agent depend on the action value ${\hat{R}}_{a}$. The movements of infants appear random and occasional, whereas those of their parents are voluntary. Thus, we model the selection and performance of infant actions by a Boltzmann equation

(17) $$\pi_{\text{child}}(t)=\frac{\text{exp}\left({\hat{R}}_{a}(t)/\tau \right)}{c+\text{exp }\left({\hat{R}}_{a}(t)/\tau \right)},$$

where *c* is the initial probability that an action is taken. The temperature parameter τ determines the randomness of action selection.

### 2.2. Interaction experiment with a virtual robot

To validate its applicability in real communication, the proposed model was implemented in a virtual agent. The virtual agent communicates with a human experimenter who mimics parent-like facial expressions and behavior.

**Figure 2:**
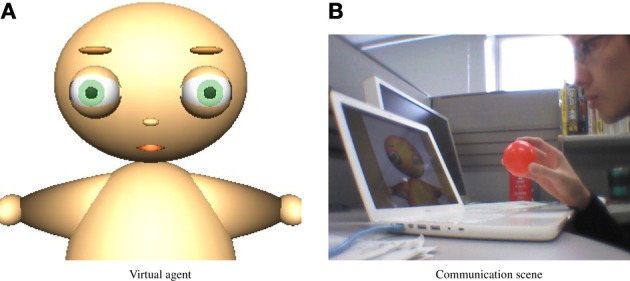
**Experimental setup. (A)** Virtual agent. **(B)** Communication scene.

The experimental setup is shown in Figure 2. Displayed on a laptop computer, the virtual agent (Figure 2A) observes facial expressions and behavior of the experimenter by a USB camera attached to the top of the laptop display.

**Figure 3:**
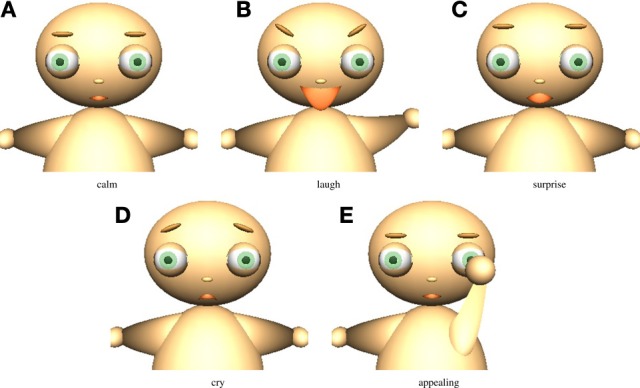
**Facial expressions and appealing behavior of the virtual agent. (A)** Calm, **(B)** laugh, **(C)** surprise, **(D)** cry, and **(E)** appealing.

The virtual agent displays four types of facial expressions depending on its emotional state. It also exhibits an appealing behavior by arm movement. The facial expressions and appealing behavior are shown in Figure 3.

The experiment was undertaken in two phases. In the first (learning) phase, the virtual agent learns the relationship between the experimenter's facial expressions and its corresponding emotional states and constructs a layered network for behavior recognition. In the second (interaction) phase, the virtual agent communicates with the experimenter.

In the learning phase, information for emotional estimation and behavior detection is processed from camera images. During emotional estimation, the estimated emotional state of the experimenter, ***e***_other_, is output from the camera image, ***x***. The facial area in the captured image is extracted by the facial recognition algorithm in OpenCV (Bradski, 2000), converted to a gray scale image of size 128 × 128 pixels, and binarized by a specified threshold. In the learning phase, a certain number of facial images, ***I***_*i*_, is recorded and each is stored with its corresponding emotional state, ***e***_*i*_. The correspondence between the emotions of the virtual and human agents is learned by imitation (Watanabe et al., 2007); that is, the human agent imitates the facial expressions of the virtual agent when presented with a stimulus such as a blue object or keyboard pressing (these responses of the virtual agents are pre-programmed). In the interaction phase, the input facial image ***I***_*x*_ is compared with the stored images and the best-matched facial image is selected as

(18) $$I_{\text{min}}=\arg\;\underset{I_{i}}{\min}|I_{x}-\;I_{i}{|}^{2}$$

Let ψ be the mapping function. The momentary estimated emotional state of the experimenter is calculated as

(19) $$e_{\text{now}}=\psi (I_{\text{min}}).$$

The estimated emotional state is the temporal average of the momentary estimated emotional states,

(20) $$e_{\text{other}}=(1-\delta )e_{\text{other}}+\delta e_{\text{now}}$$

where δ is an update constant.

**Figure 4:**
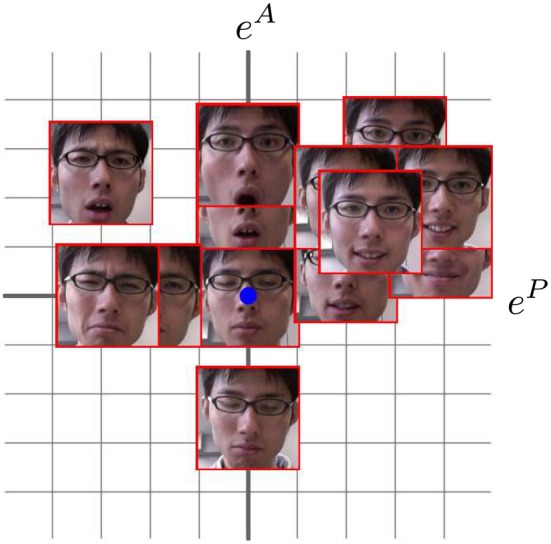
**Learned map of facial images and emotional states**.

Figure 4 shows the learned map of the facial images and their corresponding emotional states. The vertical and horizontal axes indicate the arousal and pleasure levels, respectively. For this experiment, the emotional state of the experimenter is estimated from 25 images.

Behavior recognition is achieved by a layered neural network of slow feature analysis (SFA). The SFA learning algorithm extracts the slowly changing components from the input signals and estimates the inherent information based on their statistical properties (Wiskott and Sejnowski, 2002). According to some studies, SFA exhibits stronger gesture recognition performance than existing methods such as the hidden Markov model and random forest (Koch et al., 2010).

The input to the SFA layered network is an image of the experimenter waving a red object in his hand in various directions. The learning data are 2000 steps of images. The input image (320 × 240 pixels) is segmented into the small areas of size 16 × 12 pixels. Each small area is labeled as “1” if the number of red pixels (specified by RGB content R ≥ 160G ≤ 50B ≤80) exceeds half; otherwise, it is labeled “0”. The resultant 128-dimensional vector is used to construct a state transition model. The range of the *j*-th unit in the SFA output layer, *y*_*j*_, is divided into *S*_*j*_ bins. The output signal is described by the discrete states *s*(*y*_*j*_) (*s* ∈ {1, 2, …, *S*_*j*_}). from which the state transition probability in the *j*-th output signal, *y*_*j*_, is calculated as

(21) $$p_{ss^{\prime }}^{j}=Pr\{s\left(y_{j}(t+1)\right)=s^{\prime }|s\left(y_{j}(t)\right)=s\}.$$

This state transition model is iteratively updated when a new state is observed.

The information gain of *y*_*j*_, *I*_*j*_(*t*), is calculated by the state transition model as

(22) $$I_{j}(t)=-\frac{1}{t_{a}+1}\sum_{t=t-t_{a}}^{t}\text{log }p\left(s\left(y_{j}(t+1)\right)|s\left(y_{j}(t)\right)\right).$$

From 22, the novelty of the *j*-th output signal is evaluated as

(23) $${\text{nov}}_{j}(t)=\frac{1}{1+\text{exp}\left(-m\left(I_{j}(t)-\theta \right)\right)}.$$

Finally, the novelty of the whole output signal, nov(*t*), is calculated as the average of the novelty of each output signal

(24) $$\text{nov}(t)=\frac{1}{n}\sum_{j}^{n}{\text{nov}}_{j}(t).$$

**Figure 5:**
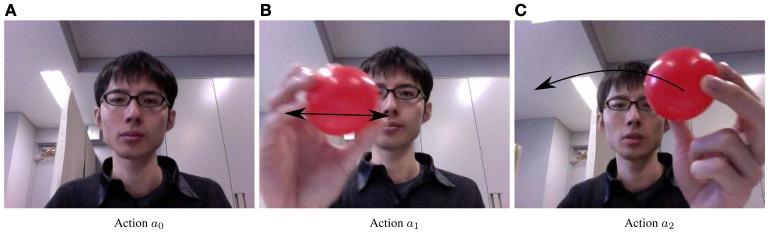
**Actions made by experimenter. (A)** Action *a*_0_. **(B)** Action *a*_1_. **(C)** Action *a*_2_.

During the interaction phase, the experimenter communicates with the virtual infant agent with a red object in his/her hand. The communication mimics the still-face paradigm experiment in developmental psychology, passing through the three phases of interaction, still face, and reunion. During the interaction phase, the experimenter looks at the camera and expresses surprise, simulating a parent seeking the attention of his/her infant. Then, when the agent similarly expresses surprise, the experimenter ensures that the arousal emotion is shared and begins laughing to the virtual agent. Throughout the interaction, the experimenter moves the red object, starting with the action patterns shown in Figure 5, and later by free motion. The persistent changes in the action pattern maintain the arousal level and the attention of the virtual infant.

During the second phase (still face), the experimenter ceases object movement and shows a blank facial expression. The possible unfamiliarity between experimenter and agent is non-problematic, because in actual still-face paradigm experiments, the still-face effect is elicited in infants meeting a person for the first time (Adamson and Frick, 2003).

During the last phase (reunion), the experimenter reverts to the interaction phase; that is, moving the red object and presenting emotional facial expressions.

**Figure 6:**
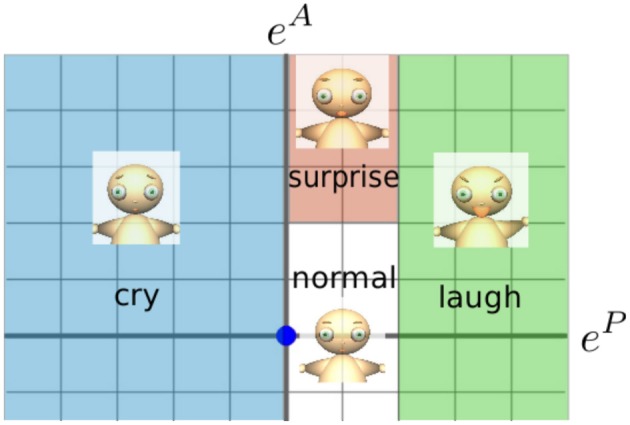
**Facial expressions and emotional states of a virtual infant agent**.

The virtual agent shows simulates laughing (*e*^*P*^ > 0.4), crying (*e*^*P*^ < 0), surprise (0 < *e*^*P*^ < 0.4, *e*^*A*^ > 0.4), and normal (otherwise). The relationship between the emotional states and the facial expressions is shown in Figure 6. The agent appeals (Figure 3E) to the experimenter based on the probability of action taken (Equation 17).

The simulated still-face paradigm experiment was conducted over the time frame of the equivalent developmental psychology experiment (Adamson and Frick, 2003); 2 min for the first interaction, 2 min for the still face, and 2 min for the reunion.

While the emotional expressions and actions of the human experimenter are continuous, they are undertaken by the virtual agent in a numerical time step (430 ms). To maintain natural interaction, the agent retains the triggered facial expression and action for 4 s. The experimenter's emotional state is estimated every 5 steps. The emotional state of the agent and all other variables are updated at each step. The novelty is evaluated after each 30-step sequence of human actions (i.e., the *t*_*a*_ is set to 30 in Equation 22). The constants and parameter settings of the infant agent are described in the next section.

### 2.3. Simulation experiment of parent–infant interaction

We suppose that parent–infant interaction is enabled by a motivation mechanism that is common to both individuals. The communication dynamics are governed by mutual attraction between the infant's and parent's motivation. In this section, the proposed model is implemented in two agents to determine whether the parent–infant interaction emerges through interplay between emotion and action in a simulation environment. We also examine how the relatedness of the infant agent changes in response to varying patterns of action and emotional expressions presented by the parent agent.

Both agents are assigned three actions, *a*_0_, *a*_1_ and *a*_2_, as shown in Figure 5. The parent agent selects its action from the repertoire when the action value is updated. If the probability of action (Equation17) exceeds a given threshold, the infant agent adopts the action taken by the parent in the previous step.

Action recognition is based on the image sequence recorded in the interaction experiment between the human and the virtual agent. When its partner performs an action, the observing agent accepts an image sequence (30 images 9 of the active agent as input. When no action of the agent is observed, the novelty of the observer decreased by a factor of λ,

(25) nov(t)=λnov(t−1)      (if no action is observed).

In emotional expression and recognition, we assume for simplicity that one agent can observe the emotion of another agent, *e*_other_, from his/her facial expression, *f*_other_ without mistakes.

We also assume that two steps of simulation time correspond to 1 s. An action is selected, and the action value, together with the emotional estimate of another agent, is updated every 5 steps. All other variables, including the emotional state, are updated at each step.

We allocated the following five conditions of facial expression and action patterns of a parent agent.

1. normal
2. still face
3. fixed action
4. random emotion/action
5. no relatedness

Under condition (1) *normal*, the parent agent behaves according to the proposed emotional system.

Under condition (2) *still face*, the parent agent adopts the still-face behavior in human-agent interaction experiments. The simulated experiment is undertaken in three phases; interaction phase (0–999 steps), still face phase (1000–1199 steps), and reunion phase (1200–1399 steps). Each phase corresponds to 2–3 min in real time. While both agents follow the proposed model during the interaction phase, the parent ceases facial expression and activity in the still-face phase. During this phase, the emotional state of the parent agent is set to *e*^*A*^ = 0 and *e*^*P*^ = 0. In the reunion phase, the parent agent recovers its emotional expression and resumes action.

Under condition (3), *fixed action*, the parent agent selects the same action, *a*_1_, while its emotional expressions are governed by the proposed model. Unlike the normal condition, in which action selection by the parent depends on the action value, ${\hat{R}}_{a}$, the fixed action arouses marginal emotion in the infant. The resulting lack of novelty perceived by the infant reduces the relatedness.

Under condition (4), *random emotion/action*, the parent expresses random emotion expressions and performs actions randomly. The arousal and pleasure values are randomly selected from −1 to 1. Among the three-action repertoire, each action is selected with equal probability. While emotions are continuously shared between the parent and infant agents under normal conditions, emotional sharing is interrupted under this condition.

Under condition (5), *no relation*, the relatedness of the parent agent is not updated (and remains fixed at 0). This condition enables the observation of how relatedness between the agents affects their emotional sharing.

The parameters and coefficients used in this experiment are listed in Tables 1–4.

**Table 1:** **Parameters of emotional elements**.

**Parameter**	**Explanation**	**Parent/Infant**
*m*	Coefficient of information gain for novelty	100
θ	Threshold of information gain for novelty	0.9
μ	Decay constant for relatedness	0.006
ν	Coefficient of vector similarity for relatedness	0.025
σ	Coefficient of similarity for stress	5

**Table 2:** **Parameters of emotional change**.

**Parameter**	**Explanation**	**Parent**	**Infant**
α^*A*^	Coefficient of novelty for arousal reward	0.8	0.5
β^*A*^	Coefficient of stress for arousal reward	0	2
α^*P*^	Coefficient of relatedness for pleasure reward	0.8	0.45
β^*P*^	Coefficient of expectancy for pleasure reward	0	40
γ	Coefficient of emotional contagion for pleasure reward	0.6	1
η_*R*_*e*__	Coefficient for update of emotional state	0.03	

**Table 3:** **Parameters of action motivation**.

**Parameter**	**Explanation**	**Parent**	**Infant**
ω	Coefficient of relatedness for motivational value	0.3	–
ρ	Weight of motivational value for action reward	1	0
η_*R*_*a*__	Coefficient of action value update	0.6	
ε	Probability that parent selects random action	0.1	–
*c*	Initial constant of action selection of infant	–	4
τ	Temperature constant of action occurrence probability of infant	–	0.3

**Table 4:** **Other system parameters**.

**Parameter**	**Explanation**	**Parent/Infant**
*n*	Number of input signals for novelty detection	20
*S*_*j*_	Number of bins in input signals for novelty detection	20
η_*e*_	Coefficient for emotion estimation	0.05

## 3. Results

### 3.1. Experimental results in interaction experiment with virtual robot

**Figure 7:**
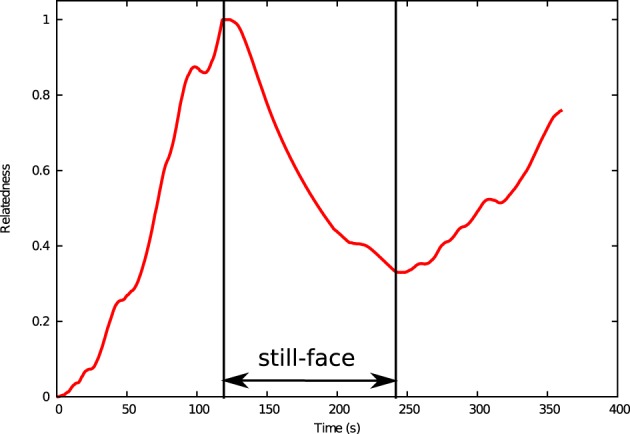
**Relatedness of virtual agent as a function of time in the simulated still-face experiment**.

Throughout the 6-min interaction period, the virtual agent completed 828 calculation steps. Figure 7 shows the temporal profiles of relatedness during the interaction. Throughout the first interaction phase, the relatedness increases to its maximum value 1.0 in 118 s. The relatedness declines throughout the still-face phase (from 120 to 240 s) is minimized (0.33) at 247 s and recovers throughout the reunion phase (after 240 s) when normal interaction is resumed.

**Figure 8:**
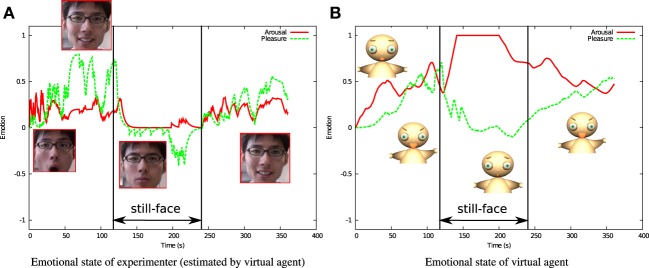
**Emotional state of experimenter (estimated by virtual agent) **(A)** and virtual agent **(B)****.

Figure 8A shows the emotional state of the experimenter estimated by the infant agent. While the experimenter shows a positive emotional state in the interaction and reunion phases, its arousal and pleasure value fall to 0 during the still-face phase.

**Figure 9:**
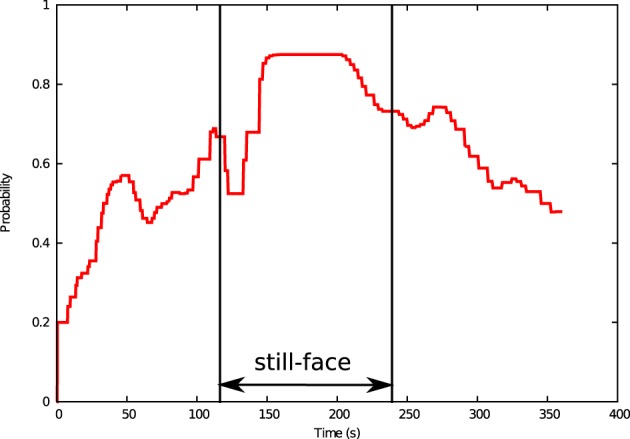
**Probability of agent action as a function of time in the simulated still-face experiment**.

Figure 8B shows how the emotional state of the infant virtual agent changes over time. During the first interaction phase, positive emotion continues, and the pleasure level increases with increasing relatedness. Note that the arousal level suddenly escalates in the still-face phase, while the pleasure level decreases. During the reunion phase, the arousal settles around 0.5, and the pleasure recovers. Figure 9 shows the probability of action taken by the agent. This probability increases with increasing pleasure level throughout the interaction phase, but suddenly leaps in the still-face phase. This trend mirrors the appealing behavior of infants real-time still-face experiments.

### 3.2. Experimental results of simulated parent–infant interaction

**Figure 10:**
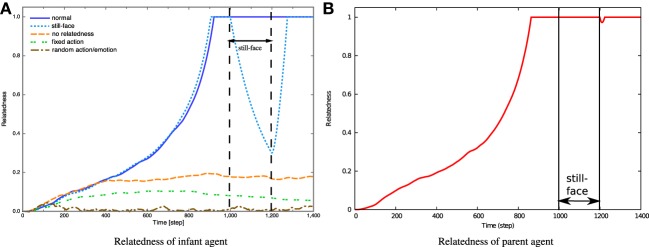
**Relatedness during simulated parent–infant interactions. (A)** Relatedness of infant agent. **(B)** Relatedness of parent agent.

Figure 10 shows the temporal dynamics of relatedness in the infant agent while interacting with the parent agent under the five conditions. While the relatedness increases to its maximum as the interaction proceeds under *normal* and *still-face* conditions, it remains low under the remaining three conditions. During the 860 steps of the interaction phase under the *sitll-face* condition (corresponding to the *normal* condition), the relatedness increases to 1. However, while the relatedness remains at 1 under normal conditions, it declines throughout the still-face phase, because the parent shows not emotional expression, and the degree of emotional sharing, sim, reduces to 0. In the reunion phase, after 1202 steps, the relatedness recovers as observed in the human–robot interaction experiment.

**Figure 11:**
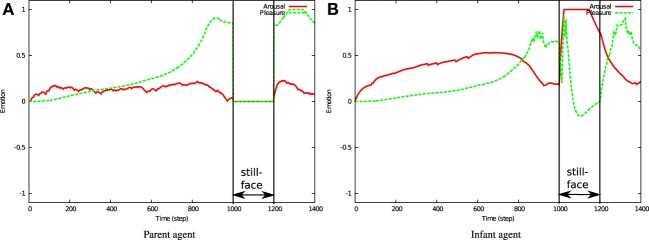
**Emotional states during simulated still-face interactions**. Red and green lines indicate the arousal and pleasure levels, respectively. **(A)** Parent agent. **(B)** Infant agent.

Figure 11 shows the emotional states of infant and parent agents. Throughout the interaction phase, the actions of the parent engage the infant agent, raising its arousal level. The increased relatedness enhances the pleasure level in both agents. During the still-face phase, both the arousal and pleasure levels of the parent agent decrease to 0 (Figure 11A). On the other hand, the resulting stress to the infant (described by Equation 10) increase its arousal level (Figure 11B). Increased arousal is accompanied by a decline in the pleasure level shortly after entering the still-face phase. This negative emotion is induced by the negative value of expectancy value (Equation 11). During the reunion phase, the arousal level of the infant decreases to pre-stress levels, and the pleasure level is recovered as relatedness is restored.

Under the *fixed action* condition, the relatedness increases to 0.1 and gradually declines to a low level. By contrast, relatedness remains low under *random action/emotion* conditions. As defined in Equation (7), relatedness is determined by the similarity of emotional states between the two agents. Throughout the interaction phase, the arousal level of both agents necessarily increases, as specified in the still-face condition. However, when the parent performs fixed actions, it stimulates no novelty in the infant. Although random actions do stimulate novelty in the infant, the randomness of the parent's emotional expressions interrupts emotional sharing, thereby reducing the relatedness under *random action/emotion* conditions.

Under the *no relatedness* condition, the relatedness of the infant agent increases up to around 0.2 during the first 400 steps and remains at 0.2 thereafter. During the interaction phase, the shared arousal emotion enhances the relatedness. Subsequently, the shared pleasure emotion further increases the similarity, sim, and thus the relatedness. However, since relatedness dominates the pleasure level, pleasure cannot increase if the parent lacks relatedness. Thus, high relatedness in the infant agent can be achieved only by the sharing of arousal emotion.

## 4. Discussion and conclusion

In our model, parent infant interactions are primarily mediated through novelty and relatedness. Novelty motivates interaction with the environment. Since the novelty value is evaluated from a pre-learned state transition model, it is increased by the perception of dynamic movement and reduced in still environments. Based on this property, the parent predicts which action will elicit higher novelty in an infant, such as moving an object. As the infant detects novelty in his/her parent's behavior, its arousal level and response frequency are enhanced. In turn, the infant responses evoke novelty, and hence arousal, in the parent. Increased arousal in both agents increases emotional sharing, and hence the relatedness, between the agents. This enhanced relatedness encourages pleasurable emotions and further emotional sharing. The simulation experiment demonstrated this positive feedback effect of mutually exchanged rewards.

In the proposed model, novelty and the state transition probabilities of other agent's actions are evaluated by SFA networks. Such networks are effective for extracting similar action structures from image sequences, because they can integrate temporarily similar information. This property of SFA networks renders them suitable for gesture recognition, where repeat observations of the same action are perturbed by human motion and lighting conditions. In fact, unvarying repeated action decreases the novelty, because the same state transition is observed.

The relatedness modeled in this paper does not consider long-term relationships. We reiterate that the still-face paradigm is applicable not only to parent–infant interactions but also to stranger–infant interactions (Adamson and Frick, 2003). Furthermore, the still-face response is absent during interactions with impersonal objects. This finding indicates that infants can relatively quickly identify whether an object/person is amenable to social interaction and can related to that object or person. Humans do not empathize with objects and other humans that fail to comply with expectation, unless relatedness is also present. Relatedness is regarded as a precursor to all social emotions, including social expectation, social contagion, and social stress. For this reason, the modeled terms of social contagion, stress, and expectation of emotional reward include multiples of relatedness.

An interesting result of the proposed model is that surprise appears first in the interaction, followed by pleasure. This is attributable to the evocation of arousal by novelty detection, which occurs regardless of relatedness, while the pleasure emotion arises only through relatedness. Thus, during in the initial interaction, when relatedness is low, arousal is elicited first. Next, as arousal is shared, the relatedness is increased, followed by pleasure, which elicits the smiling response. In this way, emotional contagion encourages further emotional sharing.

Although the parent frequently changes action during interaction phase, the frequency of change decreases as relatedness increases. High relatedness maintains the motivation at a high level and prevents the decline of the action value. Indeed, in the simulation experiment, the parent altered its actions 41 times through the interaction phase, increasing to 96 times when relatedness was set to 0. In actual human communications, this trend might signify a shift from a unidirectional form, in which a parent attracts the attention of an infant, to a bidirectional form, in which both parent and child pursue pleasurable emotions.

The simulation experiment investigated how the interaction between the parent–infant interaction changes when the relatedness of the parent agent is not updated. Under this condition, the emotional state of the parent is static, and the action patterns depend on emotional sharing with the infant. During the first phase, the arousal level increases in both parent and infant agents, increasing the relatedness and pleasure levels of the infant, while those of the parent remain fixed. In this case, because the emotional state vectors of both agents diverge, the relatedness and pleasure levels of the infant remain low. Thus, if one agent seeks relatedness and its accompanying pleasure, it must find another agent with the same goal at the same time. Baumeister and Leary (1995) proposed that human beings are fundamentally and pervasively motivated by a need to belong; that is, to form enduring interpersonal attachments. According to these authors, this need is satisfied when pleasant interactions occur within a temporally stable and enduring framework of affective concern for each other's welfare. In our simulation study, a similar reciprocal relationship between two agents was required to maintain interpersonal attachments.

In the simulation experiment, the relatedness was initialized to 0 both in both agents. In an actual interaction, the parent who establishes communication with his/her infant possesses high relatedness at the beginning of the interaction. However, if the initial pleasure value of the parent agent is set to 1, the relatedness decreases, because the pleasure level does not match that of the infant. This occurs because relatedness in the proposed model depends only on emotional similarity. This problem might be solved by including a top–down mechanism, such as a bias term, when calculating the relatedness in the parent agent. Such a term would account for the parent's desire to interact with the infant.

In this paper, the emotional state of an agent is defined in a two-dimensional plane whose aces are arousal and pleasure. This low-dimensional model of emotions has been previously adopted in robotics studies (Breazeal and Scassellati, 1999; Itoha et al., 2005; Watanabe et al., 2007). In psychology, low-dimensional models are based on descriptive taxonomies and have proven reasonably successful for describing measures of self-reported emotion and relative confusion of various facial expressions. However, the sections of brain corresponds to each dimension are not clear. Arguably, such models cannot explain selective emotional impairments (Calder et al., 2001). The difficulties in modeling emotions necessitate a direct quantitative comparison of the model with psychological experiments. Facial expressions and physiological data such as Galvanic skin response are superficial expressions of internal emotional states. In this paper, the still-face effect is qualitatively compared with the psychological still-face paradigm experiment. Although emotions appear to be dispersed within the human brain, unlike the physical sense of touch, which is located in the somatosensory area, separated areas are probably connected within the state space of emotion. In future experiments, we plan to incorporate brain mechanisms, including the relationships among brain regions related to emotion, and to compare the theoretical model with brain activities during interactions (Dumas et al., 2010).

Gaze is one of the most important challenges in extending the proposed model. Arousal is closely related to attention. In the proposed model, an agent informs its interest to another by arousal-induced actions but does not inform the item of interest. Furthermore, the parent's action value varies over time, but it is independent of sensor information. Supplied with gaze information, a parent could locate and identify the item commanding the infant's attention, which would enrich communication. For example, parental behavior such as intentionally shifting the timing of an action or showing exaggerated facial expressions after attracting the infant's attention would further enhance pleasure in the infant.

The proposed model does not explain the decrease of the infant's attention toward the parent in the still-face phase. Such behavior is thought to decrease the stress experienced by the infant (Field, 1981). If true, our model must introduce attention mechanisms for controlling emotion. Furthermore, including gaze information, we could extend our simulated interactions from dyadic interactions to triadic relationships among parent, infant, and object. Especially, joint attention, in which the infant attends to the object occupying the parent's attention or promotes the parent to attend to his/her object of interest, is an important topic in the communication of shared emotion. The learning of joint attention has already been modeled in developmental cognitive robotics (Nagai et al., 2003; Triesch et al., 2006). In future extensions of our model, we aspire to understand how higher cognitive functions such as joint attention relate to motivational behavior such as novelty and relatedness.

### Conflict of interest statement

The authors declare that the research was conducted in the absence of any commercial or financial relationships that could be construed as a potential conflict of interest.
